# Direct observation of cell cycle progression in living mouse embryonic stem cells on an extracellular matrix of E-cadherin

**DOI:** 10.1186/2193-1801-2-585

**Published:** 2013-10-31

**Authors:** Dragomirka Jovic, Asako Sakaue-Sawano, Takaya Abe, Chong-Su Cho, Masato Nagaoka, Atsushi Miyawaki, Toshihiro Akaike

**Affiliations:** Department of Biomolecular Engineering, Tokyo Institute of Technology, Yokohama, 226-8501 Japan; Brain Science Institute, RIKEN, 2-1 Hirosawa, Wako-city, Saitama, 351-0198 Japan; Laboratory for Animal Resources and Genetic Engineering, Riken CDB, Kobe, 650-0047 Japan; Research Institute for Agriculture and Life Sciences, Seoul National University, Seoul, 151-921 Republic of Korea; Department of Cell Biology, Neurobiology and Anatomy, Medical College of Wisconsin, Milwaukee, WI 53226 USA; Frontier Research Center, Tokyo Institute of Technology, 4259 Nagatsuta-cho, Midori-ku, Yokohama, 226 8501 Japan

**Keywords:** Cell cycle, Fucci, Embryonic stem cells, E-cad-Fc, Live imaging

## Abstract

Self-renewal and differentiation of embryonic stem cells are tightly coordinated with cell-cycle progression and reconstructions. However, technical approach to directly visualize single embryonic stem cells still remains challenging. Here we combined two independent systems by using artificially constructed extracellular matrix that maintains embryonic stem cells in single level with cell cycle visualization reporters to directly observe cell cycle progression. Using Fucci (fluorescent ubiquitination-based cell cycle indicator) technology and computer-assisted fluorescence microscopy we were able to visualize cell cycle progression of mouse embryonic stem cells prepared from Fucci2 knock-in mice (mES/Fucci2). Imaged mES/Fucci2 cells were plated on coverslips coated with recombinant E-cadherin-IgG Fc (E-cad-Fc). This artificial extracellular matrix effectively increases adherence of cultured cells to coverslips, which is advantageous for fluorescence imaging. mES/Fucci2 cells on the E-cad-Fc maintained the typical cell cycle of mES cells with truncated G_1_ phase and pluripotency. During time-lapse imaging, we were able to track these cells with dendritic-like cell morphology and many pseudopodial protrusions. By contrast, the cell cycle progression of mES/Fucci2 cells on mouse embryonic fibroblasts (MEFs) was not observable due to their compact aggregation. Cell cycle duration of mES/Fucci2 cells on the E-cad-Fc was 16 h. Thus, the unique properties of our immunocytochemical analysis have revealed that decline of pluripotency of the Fucci2 mES cells on the E-cad-Fc was coordinated with their differentiation.

**Electronic supplementary material:**

The online version of this article (doi:10.1186/2193-1801-2-585) contains supplementary material, which is available to authorized users.

## Introduction

Stem cells have a great potential to be used for cell therapy, treatment of human disease and development of high quality cell sources (Thomson et al. 1998; Takahashi et al. 2007; Lerou & Daley 2005). The balance between proliferation and differentiation of stem cells is carefully regulated to ensure stem cell functions while limiting cellular damage (Pietras et al. 2011). The cell cycle is regulated by both intracellular and extracellular signals in a multi-cellular context (Nurse 2000; Singh & Dalton 2009). However, the molecular regulations coordinating proliferation and differentiation still remain unclear. Embryonic stem cells generated from embryo and iPS cells represent an advantageous system to model cellular regulation because they can proliferate indefinitely and have capacity to differentiate into three germ layers. Recently, a fluorescent cell cycle indicator (Fucci) was developed to visualize dynamics of cell-cycle progression of mammalian cells both in vitro and in vivo (Sakaue-Sawano et al. 2008a; Sakaue-Sawano et al. 2008b). The Fucci technique was further extended to illuminate cell-cycle progression in developing zebrafish embryo, human embryonic stem cells (hESCs) and naïve murine stem cells (Sugiyama et al. 2009; Pauklin & Vallier 2013; Calder et al. 2013; Roccio et al. 2013; Coronado et al. 2013). However, it is still challenging to visualize cell-cycle progression of single mouse embryonic stem (mES) cells on a coverslip, because these cells aggregate into dense colonies which obstruct clear single cell visualization.

Here we have established a new embryonic stem cell line (Fucci2 mES cells) with aim to visualize cell cycle progression of mouse ES cells individually, which had been plated on recombinant E-cadherin as an artificial extracellular matrix (ECM). We were able to induced differentiation into endoderm like cells and visualized cell cycle reconstruction in early stage of differentiation accompanied by an increase in cell size. This system might be used not only for cell cycle visualization but for nuclear and cell organelles reorganization during differentiation commitment. Moreover, cells in this form might be recognized as an ideal model to investigate properties or function of individual molecules in single-molecule experiments. Previously we reported that mES cells on E-cad-Fc retained pluripotency, these cultured cells grew without colony formation and were used to generate germline-competent chimeric mice (Nagaoka et al. 2006; Nagaoka et al. 2008).

## Materials and methods

### Fucci2 mES cells

R26-mCherry-hCdt1(30/120) (Acc. No. CDB0264K) and R26-mVenus-hGem(1/110) homozygous mice (Acc. No. CDB0265K) were obtained from the Laboratory for Animal Resources and Genetic Engineering, RIKEN Center for Developmental Biology (Abe et al. 2013). Fucci2 mouse embryonic stem (mES) cells were cultured as described previously with some modifications (Nagy et al. 2003). R26-mVenus-hGem(1/110) female mice were mated with R26-mCherry-hCdt1(30/120) male mice. Fertilized eggs were cultured to the 2-cell stage and then were frozen as stocks. After thawing, 2-cell stage embryos were individually cultured in FulTrac wells in KSOM-AA medium for 24 hours in a CO2 incubator. The medium were changed to RPMI1640 containing 20% FCS (Nishioka et al. 2008; Sakaue-Sawano et al. 2013). Then, cells were plated onto 4-well plates containing a feeder layer of mitomycin-C treated mouse embryonic fibroblast (MEFs), cultured in KnockOUT DMEM (Gibco) supplemented with 10% (v/v) KnockOut Serum Replacement, 1 mM L-glutamine (Gibco), 1% non-essential amino acids (Gibco), 0.1 mM β-mercaptoethanol (Nacalai), and 1,000 units/ml ESGRO (Millipore). The cells were passaged every 3 days with Accutase (Millipore). For all imaging data cells reseeded on E-cadherin-Fc-coated (E-cad-Fc) glass bottom dish were used. MEF cells were cultured on 0.1% gelatin coated plates in D-MEM (Gibco) medium supplemented with 10% FCS, 1% L-glutamine (Gibco) and 0.5% penicillin/streptomycin (Gibco). The experimental procedures and housing conditions for animals were approved by the Animal Experimental Committees at the Institutes of Physical and Chemical Research (RIKEN) -Research Center for Allergy and Immunology (RCAI) and -Brain Science Institute (BSI), and Kyoto University school of medicine, and all animals were cared for and treated humanely in accordance with the Institutional Guidelines for Experiments using Animals.

### Construction of E-cad-Fc and preparation of E-cad-Fc-coated dishes

Expression and purification of E-cad-Fc chimera protein were described in (Nagaoka et al. 2006). In brief, E-cad-Fc fusion protein was generated using the E-cadherin extracellular domain cDNA from mouse E-cadherin full-length cDNA provided by the RIKEN BRC DNA Bank (code 1184), and mutated mouse IgG1 Fc domain cDNA (T252M/T254S). To prepare the E-cad-Fc-coated surface, purified E-cad-Fc solution was directly added to non-treated polystyrene plates. After 2 h of incubation at 37°C, plates were washed with PBS once, and then cells were seeded. Characteristic single-cell scattering morphology was observed after 24 h of incubation at 37°C.

### Fucci2 mES cell differentiation in vitro

The differentiation medium was identical as described above for Fucci2 mES cell culture medium without leukemia inhibitory factor (LIF). Different combinations of growth factors, soluble factors and small molecules were used: 10 ng/ml of activin A (R&D systems) in differentiation medium I for first three days was used. Following three days combination of 10 ng/ml activin A and 50 ng/ml basic fibroblast growth factor (bFGF; Promega) was used for differentiation medium II. In case of more differentiation induction, after day 6 we used combination of 10 ng/ml hepatocytes growth factor (HGF, Sigma), 10 ng/ml oncostatinM (OSM, Sigma) and 1 mM dexamethasone (DEX, Sigma) in differentiation medium III.

### Flow cytometry analysis of cell cycle distribution

Hoechst 33342 solution (56 μl of 1 mg/ml stock) (DOJINDO, Kumamoto, Japan) was added to a dish containing Fucci2 mES cells. After incubation for 30 min, cells were harvested and analysed using a FACSAria II (BD Bioscience, San Jose, CA). Hoechst 33342 was excited by a UV Laser at 355 nm, and its emission was collected through 405/20 BP. The data were analysed using FlowJo software (Tree Star).

### Immunofluorescence and alkaline phosphatase staining

Fucci2 mES cells were fixed with Mildform 20 N (8% formaldehyde) for 20 min. The primary antibodies used in the study include rabbit anti-Oct3/4 (Santa Cruz Biotechnology), mouse anti-SSEA1 (Santa Cruz Biotechnology), and purified mouse anti-cytochrome c (BD Pharmingen). The samples were washed three times, and incubated with appropriate secondary antibodies conjugated with Alexa Fluor 633 (Invitrogen) at room temperature for 1 h. After three times washing samples were examined using oil objective confocal microscope FV1000D (Olympus) equipped with 488 nm (argon), 559 nm (laser diode), and 635 nm (laser diode) laser lines. Alkaline phosphatase activity was revealed using the alkaline phosphatase substrate kit (Sigma-Aldrich) according to manufacturer’s instructions.

### Imaging of cultured cells

Cells were grown on 35-mm glass-bottom dishes in undifferentiated or differentiated Knock-out D-MEM. Cells were subjected to long-term, time-lapse imaging using a computer-assisted fluorescence microscope (Olympus, LCV110) equipped with an objective lens (Olympus, UAPO 403/340 N.A. = 0.90), a red LED (620 nm), a CCD camera (Olympus, DP30), differential interference contrast (DIC) optical components, and interference filters. For fluorescence imaging confocal microscope FV1000-D (Olympus, Tokyo, Japan) was used. The LED lamp 505 nm and 590 nm was used with two filter cubes, one with excitation (562/40) and emission (641/75) filters for observing mCherry fluorescence, and the other with excitation (510/10) and emission (542/27) filters for observing mVenus fluorescence. For DIC imaging, the red LED was used with a filter cube containing an analyzer. Image acquisition and analysis were performed by using MetaMorph 7.0 software (Molecular Device, LLC) and FV10-ASW 3.0 software (Olympus).

## Results and discussion

### Establishment and characterization of Fucci2 mouse embryonic stem (mES) cells

**Figure 1:**
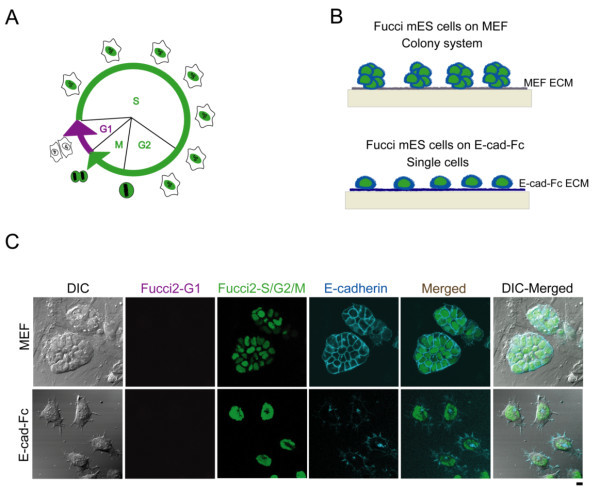
**Fucci2 probes of mES cell cycle on MEF and E-cad-Fc.** A fluorescent probe that labels individual G_1_ phase nuclei in red and S/G_2_/M phase nuclei in green **(A)**. Schematic representation of Fucci2 mES cells seeded on MEF and E-cad-Fc **(B)**. Real imaging of Fucci2 mES cells on MEF in colonies formation and E-cad-Fc with singly scattered cells using confocal microscope FV1000D **(C)**. Scale bar: 10 μm.

Fucci2-expressing mES cells were derived from blastomeres (Abe et al. 2013). These cells exhibited either red (mCherry) or green (mVenus) fluorescence in their nuclei (Sakaue-Sawano et al. 2011). The cells from 2-cell stages were grown in FullTrac wells and video-recorded (Additional file 1: Video S1). Figure 1A shows that the pluripotent ES cells derived from blastocysts of mouse maintained typical cell cycle of ES cells with shortened G_1_ phase, which was first detected in the morula period and rapidly disappeared. The morphologies of Fucci2 mES cells cultured on MEF layers and E-cadherin-Fc (E-cad-Fc) are graphically represented in Figure 1B. The Fucci2 mES cells on E-cad-Fc were scattered as reported previously (Nagaoka et al. 2006). On the other hand, the Fucci2 mES cells on the feeder layer (MEF cells) formed packed and tight colonies. In both cases, the red fluorescence of mCherry from Fucci2 mES cells was not detectable due to fast cell-cycle transition, whereas the green fluorescence of mVenus was predominant. Figure 1C shows actual images of the morphologies of Fucci mES cells on MEF and E-cad-Fc after 12 h. ES cells grown on either MEF or E-cadherin will be used for further comparison. Time lapse imaging of Fucci2 mES cells on E-cad-Fc was performed for 3 days at 30 min intervals to follow the fate of individual cells in proliferation stage using a computer-assisted fluorescent microscopy system (LCV 110, Olympus, Tokyo, Japan). Fucci2 mES cells on E-cad-Fc showed dendritic-like cell morphology with many pseudopodial protrusions and flexible movement (Additional file 2: Video S2). By contrast, Fucci2 mES cells on MEF were not individually observed due to dense colony formation (Additional file 3).

### Pluripotency of Fucci2 mES cells

**Figure 2:**
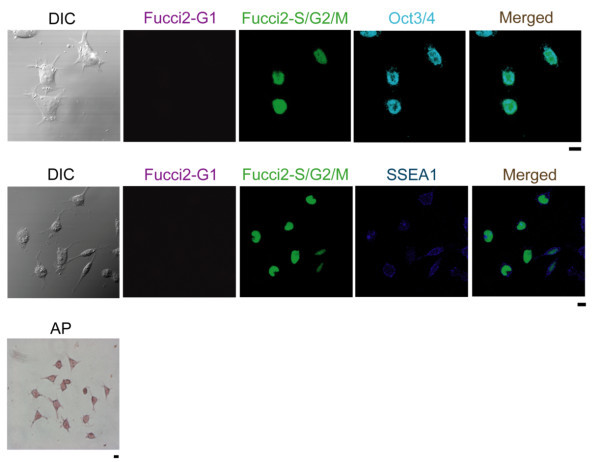
**Characterization of Fucci2 mES cell lines.** Immunosfluorescent staining of Fucci2 mES cells for expression of Oct3/4, SSEA1, and staining for alkaline phosphatase (AP) activity. Data was analyzed by confocal microscope FV1000D. Scale bar: 10 μm.

Immunohistochemistry was performed to confirm the expression of pluripotency markers in mES cells prepared from the Fucci2 Knock-in mouse line as shown in Figure 2. These cells exhibited immunosignals for Oct3/4 and SSEA1 as well as alkaline phosphatase (AP) activity, suggesting maintenance of self-renewal ability of Fucci2 mES cells.

### Cell cycle period of Fucci2 mES cells

**Figure 3:**
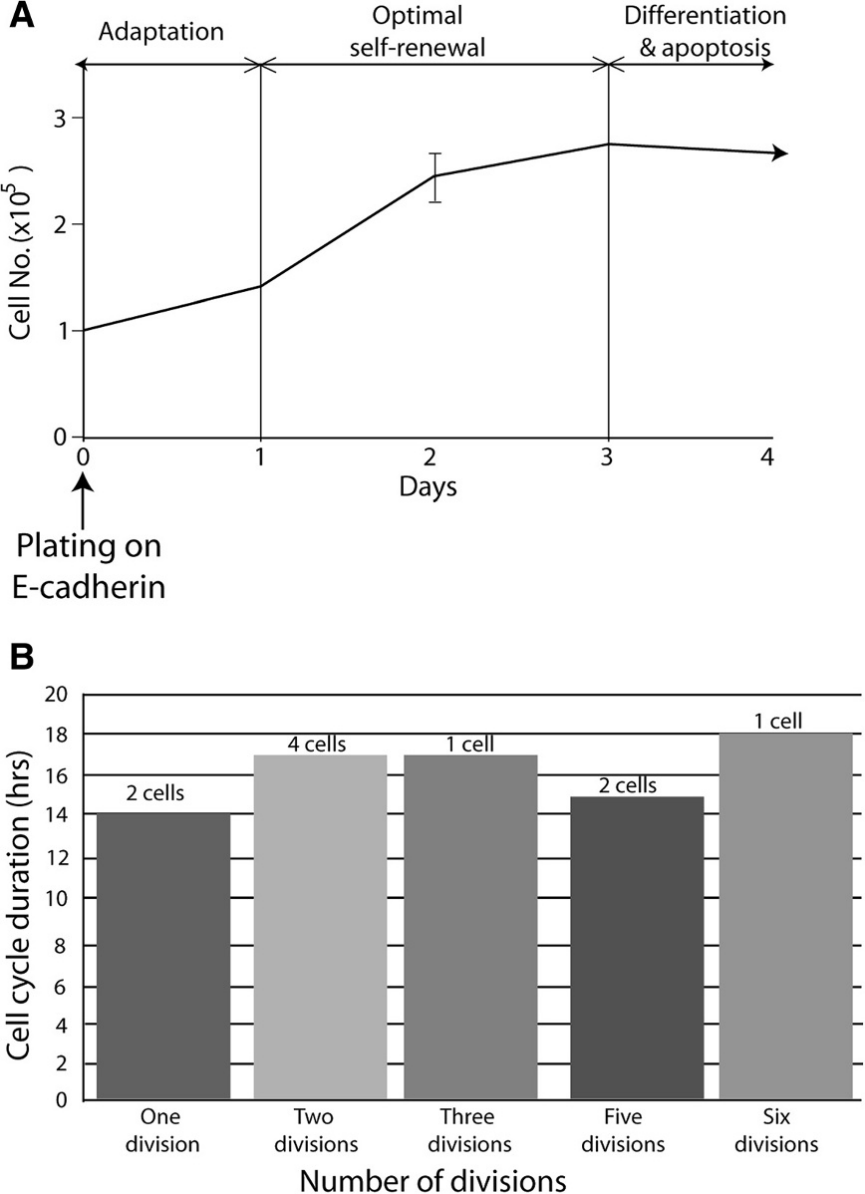
**Population doubling time and cell cycle duration of Fucci2 mES cells.** Fucci2 mES cells were plated on E-cad-Fc at a density of 1 × 10^5^ cells in 35 mm dish **(A)**. Cell numbers were counted at each day (2 to 3 replicates) in two independent experiments using a Trypan blue exclusion assay. Data are mean ± SD, n = 3. Cell cycle duration of individual Fucci2 cells was measured by time lapse video microscopy (LCV110-Olympus). Histogram represents the duration of 29 individual cells for 72 hr **(B)**.

**Figure 4:**
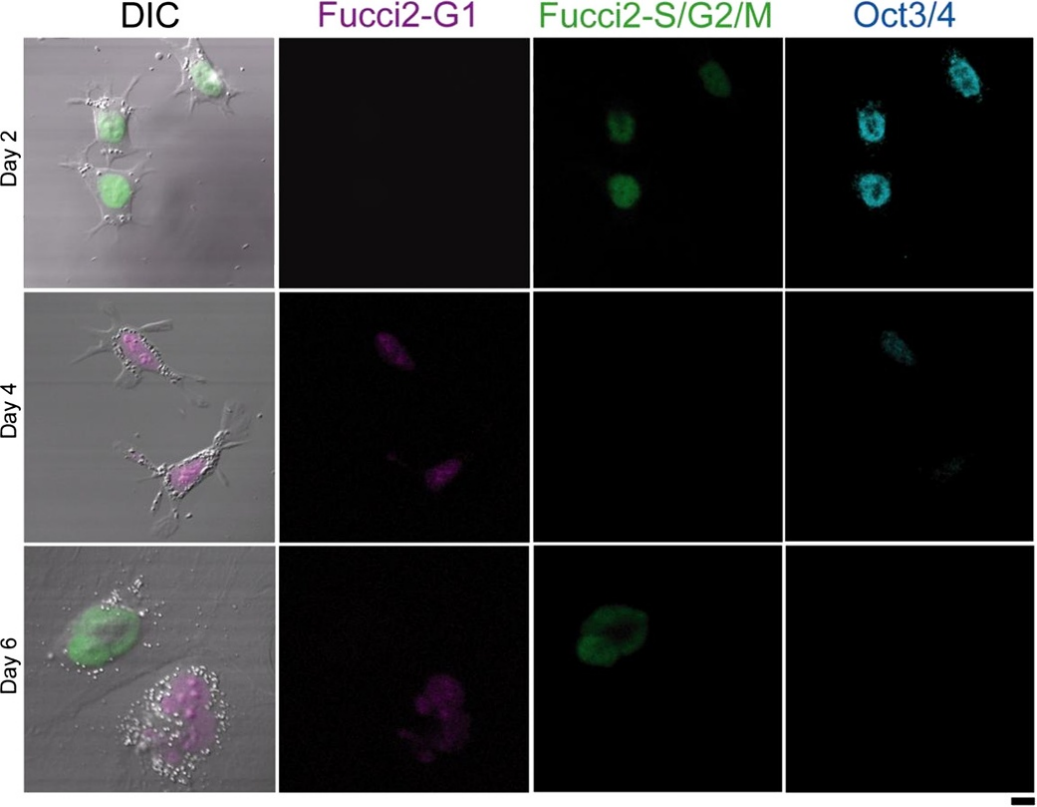
**Expression of Oct3/4 marker in Fucci2 mES cells.** Immunofluorescent detection of Oct3/4 in undifferentiated Fucci2 mES cells on E-cad-Fc at day 2, 4 and 6 of culture. Scale bar: 10 μm.

We counted numbers of Fucci2 mES cells on E-cad-Fc, after plating at a certain density in the presence of leukemia inhibitory factor (LIF). The cell number moderately increased and reached a plateau at 4 days (Figure 3A). Time-lapse imaging of individual Fucci2 mES cells on the E-cad-Fc was conducted to measure precise cell cycle period using a computer-assisted fluorescence microscopy system. Figure 3B shows the number of divisions of Fucci2 mES cells on E-cad-Fc according to the cell cycle period measured during 10 and 24 h post plating. Their total cell cycle period (median value) was 16 h. The distribution of the self-renewing Fucci2 mES cells was analysed by flow cytometry (data not shown). Cells were also immunostained to determine whether the variations in pluripotency were paralleled to changes in the expression of differentiated marker. As shown in Figure 4, the expression levels of Oct3/4 in Fucci2 mES cells on the E-cad-Fc were 99, 70, and 10% at day 2, 4 and 6, respectively, suggesting that there was an optimal growth phase at day 2 (Additional file 4: Figure S1).

### Visualization of real time dynamic imaging of Fucci2 mES cells in differentiated cell fate

Fucci2 mES cells on E-cad-Fc in the presence of activin A and bFGF as inducing agents were visualized over 5 days of differentiation to confirm derivation of endoderm progenitor cells using computer-assisted fluorescent microscope as shown in Additional file 5: Video S3. As shown in the video, cell cycle progression was easily detected in singly scattered cells through differentiation pattern. During the early stage of differentiation, cell cycle restructuration was confirmed by the presence of mCherry-positive cells accompanied by an increase in cell size and a decrease in proliferation rate.

## Conclusion

By applying the Fucci technique to mouse embryonic stem (mES) cells that grew on E-cad-Fc, we successfully visualized their cell cycle progression with sufficiently high spatial resolution. Although mES cells have strong tendency to form colonies, they can be cultured with full dissociation on the substratum without losing their pluripotency. Thus, by implementing this E-cad-Fc technique, it will be possible for researchers to carry out time-lapse imaging of individual embryonic stem cells in order to better understand spatiotemporal regulation of a variety of cellular events during the determination of cell differentiation direction and their differentiation into certain somatic cells. Moreover, it will be possible to characterize nuclear and cytoskeletal organization of pluripotent and lineage-committed cells in more detail, as well as to unravel molecular mechanism by which cell fate decision are controlled by the cell-cycle machinery.

## Electronic supplementary material

Additional file 1: Video S1: Time-lapse imaging of Fucci2 mES cells during blastocyste formation. Cells from 2 cell stages were seeded on glass-bottom dish and time-lapse imaging was performed using LCV110 microscope (Olympus). Images were acquired every 32 min. Playback speed is 1000 × real time and total imaging time is 74.5 h. (WMV 2 MB)

Additional file 2: Video S2: Time-lapse imaging of proliferation of Fucci2 mES cells on E-cad-Fc. Fucci2 mES cells were grown on glass-bottom dish covered with E-cad-Fc and time-lapse imaging was performed using LCV110 microscope (Olympus). Images were acquired every 30 min. Playback speed is 13,000 × real time and total imaging time is 20 h. Scale bar: 100 μm. (AVI 3 MB)

Additional file 3: **Time-lapse imaging of proliferation of Fucci2 mES cells on mouse embryonic fibroblast (MEF).** Fucci2 mES cells were grown on glass-bottom dish covered with MEF and time-lapse imaging was performed using LCV110 microscope (Olympus). Images were acquired every 30 min. Playback speed is 10,000 × real time and total imaging time is 23.3 h. Scale bar: 100 μm. (WMV 15 MB)

Additional file 4: Figure S1: Cells on E-cad-Fc positive for Oct3/4 at day 2, 4 and 6. Expression of Oct3/4 in mES cells on E-cad-Fc was immunostained and positive cells were counted at each day in two independent experiments. Data are mean ± SD, n = 3. (TIFF 92 KB)

Additional file 5: Video S3: Time-lapse imaging of Fucci2 mES cells on E-cad-Fc in differentiation process fate. Fucci2 mES cells were grown on glass-bottom dish coated with E-cad-Fc and visualized during process of differentiation. Time-lapse imaging was performed using LCV110 microscope (Olympus). Images were acquired every 30 min. Playback speed is 13,000 × real time and total imaging time is 115 h. Scale bar: 10 μm. (WMV 15 MB)
