# Supplementary figures and images for: Direct observation of cell cycle progression in living mouse embryonic stem cells on an extracellular matrix of E-cadherin
Source: Springerplus. 2013 Oct 31;2:585. doi: 10.1186/2193-1801-2-585 (PMC4320234; doi:10.1186/2193-1801-2-585)

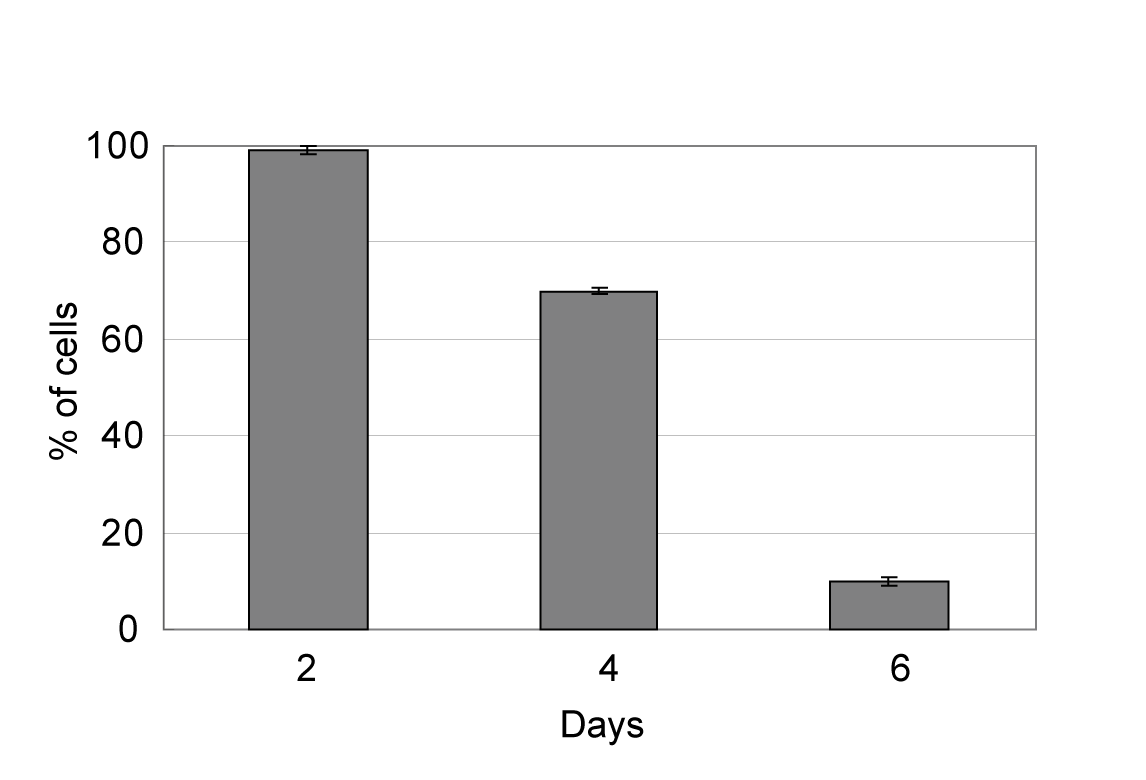

Supplement: Supplementary file 4 — Additional file 4: Figure S1: Cells on E-cad-Fc positive for Oct3/4 at day 2, 4 and 6. Expression of Oct3/4 in mES cells on E-cad-Fc was immunostained and positive cells were counted at each day in two independent experiments. Data are mean ± SD, n = 3. (TIFF 92 KB) [file 40064_2013_1438_MOESM4_ESM.tiff]
